# Mealtime caregiving approaches and behavioral symptoms in persons living with dementia: a longitudinal, observational study

**DOI:** 10.1186/s12912-021-00621-3

**Published:** 2021-06-21

**Authors:** Ji Yeon Lee, Kyung Hee Lee, Eleanor S. McConnell

**Affiliations:** 1 College of Nursing and Brain Korea 21 FOUR Project, Yonsei University, Seoul, South Korea; 2 Yonsei University College of Nursing and Mo-Im Kim Nursing Research Institute, 50-1 Yonsei-Ro, Seodaemun-Gu, Seoul, South Korea; 3 Geriatric Research, Education and Clinical Center (GRECC), Department of Veterans Affairs (VA) Medical Center, Duke University School of Nursing, Durham, NC USA

**Keywords:** Behavioral symptom, Caregiving approach, Mealtime, Person-centered behavior, Persons living with dementia, Task-centered behavior

## Abstract

**Background:**

Behavioral symptoms during mealtime can prohibit persons living with dementia from obtaining sufficient nutrition. However, little research has examined the relationship between behavioral symptoms and caregiving approaches. This study examines this relationship and further explores which specific caregiver behaviors were related to behavioral symptoms among persons living with dementia.

**Methods:**

A secondary data analysis was performed using 86 mealtime videos from a longitudinal, observational study. The videos were repeatedly taken at months 0, 3, and 6 with 30 persons living with dementia in one of four long-term care facilities. Video coding was performed using coding schemes modified from the Cohen-Mansfield Agitation Inventory for behavioral symptoms and the Person-/Task-Centered Behavior Inventory for caregiving approaches. Coding schemes for behavioral symptoms consisted of four categories: total duration, aggressive behavior, physically nonaggressive behavior, and verbally agitated behavior. Caregiving approaches consisted of ten-verbal/seven-nonverbal person-centered behavior codes, four-verbal/four-nonverbal task-centered behavior codes, and no-verbal/no-nonverbal interaction codes. A mixed-effect model was conducted using variables such as demographics, medical information, cognitive status, depression, function, and caregiving approaches as fixed effects, participant as a random effect, and four categories of behavioral symptoms as dependent variables.

**Results:**

The total duration of the Cohen-Mansfield Agitation Inventory was associated with no verbal response (*β* = 9.09) and task-centered verbal behavior (*β* = 8.43), specifically verbal controlling (*β* = 7.87). Physically nonaggressive behavior was associated with no verbal response (*β* = 9.36). Verbally agitated behavior was associated with task-centered nonverbal behavior (*β* = 51.29), and specifically inappropriate touch (*β* = 59.05).

**Conclusions:**

Mealtime is indispensable to dementia care for ensuring adequate nutrition and promoting personhood. Our findings revealed caregivers’ task-centered behaviors and no interaction were related to behavioral symptoms of persons living with dementia. When caregivers encounter behavioral symptoms during mealtime, it is recommended to avoid no response and task-centered behaviors, especially verbal controlling and inappropriate touch, and to promote person-centered behaviors.

## Background

The number of persons living with dementia was estimated at 50 million worldwide in 2019 [1]. In Korea, dementia affects approximately 750,000 people (10.16% of the population aged 65 and older), and 46.7% of long-term care residents have dementia [2]. Among persons with dementia living in long-term care residences, approximately 50% have lost the ability to feed themselves due to progressive decline of cognitive and behavioral functions [3, 4].

Maintaining appropriate nutritional status among long-term care residents is an important consideration for health professionals, as malnutrition in long-term care has been documented in 30 to 60% of residents [5]. Persons living with dementia are at a particularly high risk for malnutrition because they often develop dysphagia, the inability to eat independently, and dementia-related behavioral symptoms such as agitation and aggression [6, 7]. Although a large number of persons living with dementia receive caregivers’ assistance during mealtime, mealtime difficulties often interfere with obtaining sufficient nutrition, in part due to behavioral symptoms. In order to support adequate nutrition, it is necessary to identify caregiver factors related to behavioral symptoms and mealtime difficulties among persons living with dementia.

Studies have demonstrated that caregiving approaches were an important component for improving mealtime experiences and preventing behavioral symptoms. A review paper showed that eight studies reported providing adequate feeding assistance could improve food intake [8], and an experimental study displayed food intake and feeding behaviors were different based on meal providers’ hand-feeding techniques [9]. Additionally, a qualitative study described that limited or negative social interaction between resident and caregiver might agitate residents with dementia while knowledge of residents’ preferences and abilities was essential to reduce agitation during mealtime [10]. Taken together, these studies suggest that a person-centered caregiving approach, providing care based on an individual’s preferences and needs, would improve care quality during mealtimes and, in turn, help to maintain the nutritional adequacy of persons living with dementia [11].

However, relatively few studies have investigated the influence of person-centered care on behavioral symptoms [12, 13]. Moreover, although some studies revealed person-centered care reduced behavioral symptoms among persons living with dementia when compared to standard care or task-centered care [12, 13], the association between specific caregiver behaviors and behavioral symptoms were not examined. If we could show that certain verbal or nonverbal behaviors are associated with specific behavioral symptoms during mealtime, then caregivers could avoid or promote individual behaviors to reduce behavioral symptoms and improve meal assistance, which could result in clinically meaningful improvements in nutritional status. Thus, the purpose of this study was to examine 1) whether caregiving approaches (i.e., person-centered vs. task-centered) were associated with behavioral symptoms of persons living with dementia during mealtime and 2) which specific behaviors among caregiving approaches were related to behavioral symptoms.

## Methods

### Data source and participants

This study analyzed data from a longitudinal, observational study conducted to explore the care-specific emotional expressions of persons living with dementia. A total of 30 participants were recruited from four long-term care facilities located within a 35-km radius from the research institution. The differences between the facilities were size (40 to 80 beds) and proprietary status (two private and two public facilities). The ratios of nursing care providers to resident ranged from 1:2.2 to 1:2.45. Participants were eligible for the study if they were 65 years or older, diagnosed with dementia based on criteria from the Diagnostic and Statistical Manual of Mental Disorders, fourth edition, and had a Korean Mini Mental State Examination (K-MMSE) score lower than 24. For each participant, nine videos were taken at 0, 3, and 6 months for each of the three specific care situations including mealtime, personal care, and social activity. Since three participants dropped out of the study at 3 or 6 months, 30 participants with 258 videos were produced. Of these, this present study analyzed 86 mealtime videos from 30 participants.

### Variables and measures

#### Person-level data

Person-level data, collected only at baseline from participants’ medical charts, included demographic data such as age, sex, and education level, and medical information such as comorbidities and medications. Comorbidities were categorized according to the Cumulative Illness Rating Scale-Geriatric (CIRS-G) [14]. This tool is organized into 14 categories, and for diseases present in each category, a minimum of zero and a maximum of four points can be given according to its severity. The illness severity is calculated with the total score (0–56) divided by the number of corresponding categories (0–14). All medications taken by participants were classified as cardiovascular, diabetes, dementia, psychiatric, and other medications.

#### Observation-level data

Observation-level data, collected at 0, 3, and 6 months from the parent study included participants’ behavioral symptoms, caregiving approaches, cognitive status, depression, and function using measures described below.

Participants’ behavioral symptoms were evaluated using the Korean version of Cohen-Mansfield Agitation Inventory (CMAI-K) [15, 16]. This tool is composed of 29 items and organized into three categories: aggressive behavior (e.g., hitting, grabbing, verbal aggression, and so on), physically nonaggressive behavior (e.g., repetitious mannerisms, general restlessness, and so on), and verbally agitated behavior (e.g., complaining, negativism, repetitious sentences, and so on). A higher score means more behavioral symptoms in the original tool [17].

To measure caregiving approaches during mealtime, we used a behavioral coding scheme, which contained items from the Person-Centered Behavior Inventory (PCBI) and Task-Centered Behavior Inventory (TCBI) [18, 19]. This coding scheme measured mealtime interaction between persons living with dementia and caregivers, and comprised verbal and nonverbal behaviors for each category of PCBI and TCBI. The verbal PCBI includes ten behaviors: greeting, asking the resident for help/cooperation, giving choices, assessing comfort, providing orientation, showing approval/interest/empathy, positive voice quality, and back-channel response. The nonverbal PCBI includes seven behaviors: resident-directed eye gaze, positive gestures, appropriate use of affectionate touch, assessing comfort nonverbally, adjusting to the resident’s pace, proximity, and positive facial expressions. The verbal TCBI includes four behaviors: verbal controlling (i.e., interfering/directing tone or elderspeak), interrupting, changing topics, and controlling voice quality. The nonverbal TCBI includes four behaviors: ignoring, physically controlling, inappropriate touch, and outpacing. In addition to the PCBI/TCBI, no interaction codes (i.e., no verbal response and no nonverbal response) were added to the scheme to cover the entire video because there were parts of the video where caregivers did not display any verbal/nonverbal behaviors.

Cognitive status was measured by the K-MMSE and the Korean version of Clinical Dementia Rating (K-CDR). The K-MMSE is a 30-item scale with a total of 30 points; a lower score indicates more impairment in cognition. Untestable cases were given − 1 point in the K-MMSE [20]. The K-CDR is a 6-item scale with a total of 30 points, and a higher score on it denotes more severe dementia [21]. Depression was measured using the Korean version of Cornell Scale for Depression in Dementia (K-CSDD), which consists of 19 items with a total of 38 points; a higher score implies a more depressive state, and the cut-off score is 5 [22]. Function was rated using the Korean version of Activities of Daily Living (K-ADL), which consists of seven items; a higher score indicates more dependency, and the cut-off score is 18 [23, 24].

### Procedure

After obtaining Institutional Review Board (IRB) approval, we extracted 86 mealtime videos (i.e., eating and feeding) from the parent study, which included 258 videos about three care situations (mealtime, personal care, and social activity). Since the videos contained images and voices of participants and caregivers that would allow them to be recognized, two research assistants (RAs) who managed and coded the videos were particularly well-trained on ethical issues such as confidentiality before starting the study. To ensure intra-rater/inter-rater reliability, we initially sampled 10% of the videos, and two coders coded the same video and compared results. After inter-rater reliability as measured by the kappa statistic reached 0.8, the coding process was started. To ensure reliability between coders throughout the coding process, we conducted an additional reliability check after half the videos were coded. The overall kappa coefficient for interrater reliability was 0.89, with 0.85 at the beginning and 0.93 at the midpoint. To avoid coder fatigue and improve reliability, each rater coded only one three-hour tape per session.

We used the Noldus Observer® XT software for coding employing the items from the PCBI/TCBI and CMAI as codes. Among the PCBI and TCBI, the verbal behaviors were coded using instantaneous sampling and the nonverbal behavior were coded using continuous sampling. Instantaneous sampling counts how many times each verbal behavior appeared in the entire video (frequency) as the coder assigns the code that corresponded to specific caregiver behaviors every ten seconds. With continuous sampling, the coder assigns the code of nonverbal behaviors of interest whenever they began and finished so that the total time for each behavior could be calculated in seconds (duration) [25]. When caregivers showed no behaviors, the coder put no verbal response or no nonverbal response to provide mutually exclusive and exhaustive codes. Consistent with the PCBI/TCBI coding scheme, no interaction codes were calculated as a frequency for no verbal response and duration for no nonverbal response. The participant’s behavioral symptoms were coded using the CMAI, which was also obtained through continuous sampling. We summed the total time for each behavioral symptom observed and assigned these behaviors into the three categories of the CMAI: aggressive behavior, physically nonaggressive behavior, and verbally agitated behavior. Throughout the whole process, when the coder could not decide which category a behavior belonged to, it was determined after a discussion with another researcher at the weekly meetings.

### Statistical analysis

For statistical analysis of the frequency data, we counted the total number of times each behavior occurred in each video and grouped into person- or task-centered verbal behaviors. Then, the total frequency of person- or task-centered verbal behaviors were divided by the total time of each video (frequency per minute) since the total time of each video varied according to participants’ eating pace. Similarly, we added the total seconds of each person-/task-centered nonverbal behavior of the caregivers and behavioral symptoms of the participant and divided the total time by the total time of each video (duration per minute) to account for the difference in video length; therefore, duration per minute referred to total seconds in which a specific action appeared in 1 minute.

Statistical analysis was conducted using the STATA 16.0 software (StataCorp, College Station, Texas, USA) and consisted of the following steps: 1) descriptive analysis to understand participants’ characteristics, 2) a mixed-effect model to examine whether caregiving approaches were associated with participants’ behavioral symptoms, and 3) a further analysis to determine which particular behavior among significant PCBI/TCBI, were associated with behavioral symptoms. The mixed-effect model was chosen because the data were repeated measures and therefore nested within participants. Both time points and facility were considered in the model. We controlled for the time variable (i.e., time points) to reflect change over time in behavioral symptoms; the facility was included to account for correlation for responses from participants of the same facility. Among various mixed-effect models, we used multilevel mixed-effects tobit regression fixing the lower limit at the minimum value of the dependent variable because behavioral symptom measures were continuous variables and left-censored with many zero values [26].

## Results

The mean duration of videos was 17.58 ± 8.17 min. Table 1 shows a descriptive summary of the person-level and observation-level data. Participants had a mean age of 85.63 ± 6.67 years and were mostly female (93.33%). The mean CIRS-G score was 4.50 ± 2.74 and the severity score was 1.75 ± 0.71. The number of medications taken was 6.83 ± 3.50 and 93.33% of participants were taking dementia medications.

**Table 1:** Person-level and observation-level data

Variables	Mean (SD)	n (%)
Person-level data (*n* = 30)		
Age	85.63 (6.67)	
Sex		
Female		28 (93.33)
Male		2 (6.98)
Education level		
< Middle school (6 years of education)		16 (53.33)
> Middle school		14 (46.67)
CIRS-G		
Total score	4.50 (2.74)	
Severity score^a^	1.75 (0.71)	
Total number of medications	6.83 (3.50)	
Taking dementia medication		
Yes		28 (93.33)
No		2 (6.67)
Observation-level data (*n* = 86)		
Participants		
MMSE score	2.81 (5.18)	
CDR score	16.42 (2.24)	
CSDD score	3.72 (3.44)	
ADL score	18.51 (2.21)	
CMAI		
Total duration	7.94 (27.62)	
Aggressive behavior	0.08 (0.27)	
Physically nonaggressive behavior	6.20 (27.43)	
Verbally agitated behavior	1.65 (4.41)	
Caregivers		
Person-centered behavior		
Verbal behavior^b^	0.56 (0.72)	
Nonverbal behavior^c^	0.10 (0.13)	
Task-centered behavior		
Verbal behavior^b^	0.35 (0.63)	
Nonverbal behavior^c^	0.04 (0.08)	
No interaction		
No verbal response^b^	3.36 (2.68)	
No nonverbal response^c^	0.91 (0.44)	

*Note*. *ADL* Activities of Daily Living, *CDR* Clinical Dementia Rating, *CIRS-G* Cumulative Illness Rating Scale-Geriatric, *CMAI* Cohen-Mansfield Agitation Inventory, *CSDD* Cornell Scale for Depression in Dementia, *MMSE* Mini Mental Status Exam

^a^ Total CIRS-G score / total number of categories endorsed; ^b^ Frequency per minute; ^c^ Second per minute

Observation-level data included the values from three observation points at 0, 3, and 6 months. The mean score was 2.81 ± 5.18 in MMSE and 16.42 ± 2.24 in CDR, which indicated that most of the participants had a severe impairment in cognition. The mean score was 3.72 ± 3.44 in CSDD and 15% of participants had a depressed state. The mean ADL was 18.51 ± 2.21 and 73% of participants had impaired physical function in their ADL. The mean duration of behaviors coded by the CMAI items was 7.94 ± 27.62 sec/min. In case of sub-categories of CMAI, the mean duration was 0.08 ± 0.27 sec/min in aggressive behavior, 6.20 ± 27.43 sec/min in physically nonaggressive behavior, and 1.65 ± 4.41 sec/min in verbally agitated behavior.

In the case of PCBI, the mean was 0.56 ± 0.72 frequency/min in the verbal behaviors and 0.10 ± 0.13 sec/min in the nonverbal behaviors. In the case of TCBI, the mean was 0.35 ± 0.63 frequency/min in the verbal behaviors and 0.04 ± 0.08 sec/min in the nonverbal behaviors. The means of no verbal response and no nonverbal response were 5.36 ± 2.68 frequency/min and 0.91 ± 0.44 sec/min, respectively.

### Caregiving approaches associated with behavioral symptoms

Bivariate analysis was performed for total duration and three subcategories of the CMAI (i.e., total duration, aggressive, physically nonaggressive, and verbally agitated behavior). Each of the four CMAI categories was significantly related to different independent variables, but ADL, MMSE, and taking dementia medication had significant associations in most of the categories. Other medications (e.g., cardiovascular, diabetes, and psychiatric medications) did not have significant association with the CMAI (result not shown). Based on this bivariate analysis and previous literature, ADL, MMSE, and taking dementia medication were set as covariates. Among these covariates, taking dementia medication was not included in the aggressive behavior model because all participants with aggressive behavior were taking dementia medication (Table 2). By controlling these covariates and allowing the participant as a random effect, variables from caregiving approaches (i.e., person-centered verbal/nonverbal behaviors, task-centered verbal/nonverbal behaviors, and no verbal/nonverbal response) were used as independent variables in the multilevel mixed-effect regression.

**Table 2:** Caregiving approaches associated with behavioral symptoms of persons living with dementia during mealtime

	CMAI total duration^a^			Aggressive behavior^a^			Physically nonaggressive behavior^a^			Verbally agitated behavior^a^		
Variables	Coeff	SE	*P*-Value	Coeff	SE	*P*-Value	Coeff	SE	*P*-Value	Coeff	SE	*P*-Value
ADL	−0.08	1.05	.939	0.03	0.19	.863	−0.47	1.60	.767	−0.31	0.40	.444
MMSE	−0.37	0.38	.329	−0.07	0.06	.229	−0.97	0.66	.140	−0.12	.014	.382
Taking dementia medication	−32.52	12.66	.010	–	–	–	− 108.48	49,207.54	.998	−1.22	4.39	.781
Caregivers’ approaches												
Person-centered behavior												
Verbal behavior	8.63	4.71	.067	−5.24	5.32	.325	7.79	6.41	.224	−1.44	2.15	.503
Nonverbal behavior	−6.80	26.13	.795	26.83	31.45	.394	−18.99	33.95	.576	10.64	10.73	.321
Task-centered behavior												
Verbal behavior	8.43	4.10	.040	−5.44	5.33	.308	8.32	4.98	.095	−1.27	1.99	.524
Nonverbal behavior	42.19	26.16	.107	28.85	31.81	.364	0.93	32.07	.977	51.29	10.99	<.001
No interaction												
No verbal response	9.09	3.80	.017	−5.30	5.31	.318	9.36	4.59	.042	−1.95	1.90	.304
No nonverbal response	1.64	22.73	.942	28.92	31.89	.365	0.37	27.57	.989	6.47	9.46	.494
Time												
Baseline	Ref											
3 month	2.40	2.07	.246	−0.10	0.33	.762	.012	3.15	.969	−0.01	0.96	.995
6 month	−1.36	2.34	.562	0.56	0.32	.086	−0.04	3.36	.991	−1.55	1.05	.139

*Note*. *ADL* Activities of Daily Living, *Coeff* Coefficient, *MMSE* Mini Mental Status Exam

^a^Analysis included dummy variables for study facilities to control for their effects, but output was suppressed to protect confidentiality.

Table 2 shows the result of the multilevel mixed-effect regression. The variables associated with CMAI total duration were task-centered verbal behavior (*β* = 8.43, *p* = .040), no verbal response (*β* = 9.09, *p* = .017), and taking dementia medication (*β* = − 32.52, *p* = .010). More specifically, participants displayed more behavioral symptoms if the caregivers spoke in a task-centered manner and showed no verbal response, and if the participants were not taking dementia medications. There was no associated factor in case of aggressive behavior. Physically nonaggressive behavior was associated with no verbal response (*β* = 9.36, *p* = .042); verbally agitated behavior was associated with task-centered nonverbal behavior (*β* = 51.29, *p* < .001). In other words, participants showed physically nonaggressive behavior when there was no verbal response and showed verbally agitated behavior when the caregivers acted in a task-centered way.

### Specific caregiver’s behavior associated with behavioral symptoms

Using variables that showed significance in the multilevel mixed-effect regression, further analysis was performed to investigate which specific caregiver behaviors were associated with participants’ behavioral symptoms (Table 3). After adjusting for ADL dependence, MMSE scores, facility, and use of dementia medications, both verbal controlling behaviors by the caregiver (*β* = 7.87, *p* < .001) and no verbal response from the caregiver were significantly associated with a greater total duration of CMAI behaviors (*β* = 8.89, *p* < .001). With respect to the subcategory of the CMAI, inappropriate touching by the caregiver was significantly related to verbally agitated behavior (*β* = 59.05, *p* < .001); the more frequently the caregiver touched the participant, the more verbally agitated behaviors the participant displayed.

**Table 3:** Specific caregiver behavior associated with behavioral symptoms of persons living with dementia during mealtime

Variables	CMAI total duration^a^			Verbally agitated behavior^a^		
	Coeff	SE	*P*-Value	Coeff	SE	*P*-Value
ADL	0.66	1.31	.618	−0.07	0.34	.843
MMSE	−0.53	0.46	.253	−0.19	.012	.098
Taking dementia medication	−32.21	15.74	.041	−1.09	3.70	.769
Specific caregivers’ approaches						
Task-centered verbal behavior						
Verbal controlling	7.87	2.23	<.001	–		
Changing topic	39.17	30.52	.199	–		
Controlling voice quality	68.35	211.15	.746	–		
No interaction						
No verbal response	8.89	0.47	<.001	–		
Task-centered nonverbal behavior						
Ignoring	–			292.89	157.11	.062
Physically Controlling	–			23.51	12.98	.070
Inappropriate touch	–			59.05	8.11	<.001
Outpacing	–			11.24	39.24	.775
Time						
Baseline	Reference					
3 month	2.51	2.51	.318	−0.22	0.90	.807
6 month	−1.09	2.81	.698	−1.10	1.01	.274

*Note. ADL* Activities of Daily Living, *CMAI* Cohen-Mansfield Agitation Inventory, *Coeff* Coefficient, *MMSE* Mini Mental Status Exam

^a^Analysis included dummy variables for study facilities to control for their effects, but output was suppressed to protect confidentiality.

## Discussion

Eating well is a complex process, which can be influenced by several factors [27, 28]. Among several factors, we focused on mealtime experience, particularly on the relationship between caregiving approaches and behavioral symptoms of persons living with dementia. In general, task-centered caregiving approaches were significantly associated with participants’ behavioral symptoms, in accordance with a previous study, which reported that task-centered caregiver behaviors were more likely to precede behavioral symptoms [13]. For persons living with dementia, mealtimes can be perceived as positive events where hunger is relieved in a pleasant environment that allows for social stimulation or as negative events, times when they are forced to do tasks that are unpleasant or uncomfortable. When the caregiver behaves in a task-centered manner, it could increase the Behavioral and Psychological Symptoms of Dementia (BPSD) because task-centered behaviors do not satisfy or reduce the physiological and psychosocial unmet needs of persons living with dementia for relief of hunger or varied stimulation throughout the day [29–31]. Although the importance of person-centered care has been emphasized and applied in dementia care, it is still reported that actual dementia care is work-oriented and uses medical management styles for behavioral symptoms. This study adds more evidence that the task-centered approach is not an effective way to help persons living with dementia who manifest behavioral symptoms [32]. It is important to understand behavioral symptoms as a method that persons living with dementia use to communicate unmet needs rather than viewing these symptoms as disruptive behaviors. Therefore, health professionals need to develop competency in providing person-centered rather than task-centered care.

Our study revealed that specific caregiver behaviors were closely related to specific behavioral symptoms. When caregivers engaged in task-centered behaviors and also made verbally controlling statements, total behavioral symptom duration increased. Likewise, inappropriate touch by caregivers was related to verbally agitated behavior. Although a prior qualitative study suggests that specific caregiver behaviors influence behaviors of persons living with dementia, to the best of our knowledge, our study was the first study to examine this quantitatively using video-based observation. Our findings are consistent with those of a qualitative study [33] that found that negative caregiver actions such as confronting, persuading, or bursting out in anger tended to induce BPSD, whereas positive caregiver actions such as acknowledging and responding to patients tended to reduce BPSD. Caregivers often use verbal communication or nonverbal touch behavior to induce the resident’s response when the resident refuses the caregiver’s instruction or guidance; however, persons living with dementia may misunderstand those intentions. Another prior study also stated that excessive or ambiguous stimuli can result in mealtime agitation [10]. Since there is scarce research focused on specific caregiver behaviors and participants’ behavioral symptoms, future research is needed to confirm such a relationship. Turning now to what was already revealed, person-centered verbal and nonverbal communication is of particular importance in dementia care; furthermore, caregivers often stated they would benefit from more education [34, 35]. The results of this study specifically show that more attention is needed to avoid certain behaviors which may cause behavioral symptoms. Thus, it is important to provide staff education programs on how to relate to, connect with, and support persons living with dementia during mealtimes that focus on person-centeredness [36].

In addition to the relationships between caregiver behaviors and resident BPSD, no interaction (e.g., no verbal response) between caregivers and residents was also related to behavioral symptoms. Previous studies reported that a lack of interaction between persons living with dementia and their surroundings were associated with BPSD [37] or resulted in mealtime agitation [10]. However, persons living with dementia were found to interact directly with the caregivers only 2.5% of the time per day [38]. Even then, except for work-related interactions, much of the interaction was carried out in complete silence, with no verbal interaction [38]. Even though more interactions between caregivers and residents were expected during mealtime, similar findings were found in this study; the total amount of interactions, whether on the PCBI or the TCBI, was 2.54 times per minute on average, whereas no interaction was found 6.52 times per minute (result not shown in tables). Obstacles such as task-focused day-to-day care, workload pressure to get the task done, and a lack of education on how to communicate with persons living with dementia might hinder caregivers from promoting interaction between caregivers and persons living with dementia [35, 38].

Collectively, it seems that a person-centered caregiving approach would achieve better results than a task-centered caregiving approach or no interaction when we encounter behavioral symptoms. Despite the lack of statistical significance, coefficient values of person-centered caregiving approach tended to have a negative relationship with behavioral symptoms. When interacting with persons living with dementia, because of their decline in linguistic ability, it is necessary to utilize verbal and nonverbal communication in an appropriate person-centered manner [39].

The study had limitations. We examined the association between caregiving approaches and behavioral symptoms, but not the causal relationship. Nevertheless, it revealed the relationship of specific caregiver behaviors (i.e., verbal controlling and inappropriate touch) to behavioral symptoms of persons living with dementia with a quantitative methodology. The sample should also be noted. The parent study gathered 258 videos from 30 participants based on the power analysis, but we extracted only 86 mealtime videos. Because of the small sample size, this study might not show that person-centered behaviors had a significant negative relationship with behavioral symptoms. Thus, we suggest a future study with a larger sample size and research design to reveal any causal relationships between caregiving approaches and behavioral symptoms. Future research is also needed to determine whether fewer behavioral symptoms improve nutritional intake and consequently improve the nutritional status of persons living with dementia.

## Conclusions

In conclusion, mealtimes are vital to dementia care because they ensure adequate nutritional status and provide an opportunity for social interaction among persons living with dementia. Therefore, it is important to provide high-quality care by aiming for person-centered care, avoiding task-centered behavior, and promoting social interaction between persons living with dementia and caregivers. This study highlights that particular attention is needed to avoid overall task-centered approaches, specific behaviors such as verbal controlling and inappropriate touch, and a lack of interaction in the context of BPSD during mealtime. Our findings may provide further insight to enhance the quality of nursing care or mealtime interventions.

## Data Availability

The datasets generated and analyzed for the current study are not publicly available due to IRB agreements, but are available from the corresponding author on reasonable request.

## Abbreviations

BPSD: Behavioral and Psychological Symptoms of Dementia
CIRS-G: Cumulative Illness Rating Scale-Geriatric
CMAI-K: Korean version of Cohen-Mansfield Agitation Inventory
IRB: Institutional Review Board
K-ADL: Korean version of Activities of Daily Living
K-CDR: Korean version of Clinical Dementia Rating
K-CSDD: Korean version of Cornell Scale for Depression in Dementia
K-MMSE: Korean Mini Mental State Examination
PCBI: Person-Centered Behavior Inventory
TCBI: Task-Centered Behavior Inventory
